# A novel microscale selective laser sintering (μ-SLS) process for the fabrication of microelectronic parts

**DOI:** 10.1038/s41378-019-0116-8

**Published:** 2019-12-30

**Authors:** Nilabh K. Roy, Dipankar Behera, Obehi G. Dibua, Chee S. Foong, Michael A. Cullinan

**Affiliations:** 1 Department of Mechanical Engineering, The University of Texas at Austin, 204 E. Dean Keeton St, Austin, TX 78712 USA; 2 NXP Semiconductors, 6501W William Cannon Dr, Austin, TX 78735 US

**Keywords:** Electrical and electronic engineering, Nanoparticles, Nanometrology, Electronic devices

## Abstract

One of the biggest challenges in microscale additive manufacturing is the production of three-dimensional, microscale metal parts with a high enough throughput to be relevant for commercial applications. This paper presents a new microscale additive manufacturing process called microscale selective laser sintering (μ-SLS) that can produce true 3D metal parts with sub-5 μm resolution and a throughput of greater than 60 mm^3^/hour. In μ-SLS, a layer of metal nanoparticle ink is first coated onto a substrate using a slot die coating system. The ink is then dried to produce a uniform nanoparticle layer. Next, the substrate is precisely positioned under an optical subsystem using a set of coarse and fine nanopositioning stages. In the optical subsystem, laser light that has been patterned using a digital micromirror array is used to heat and sinter the nanoparticles into the desired patterns. This set of steps is then repeated to build up each layer of the 3D part in the μ-SLS system. Overall, this new technology offers the potential to overcome many of the current limitations in microscale additive manufacturing of metals and become an important process in microelectronics packaging applications.

## A method for microscale metal 3D Printing

A new 3D fabrication process could greatly accelerate the manufacture of finely-detailed microelectronic devices for a plethora of applications. Additive manufacturing strategies such as 3D printing are becoming commonplace in many industries, but these are generally ill-suited for producing the tiny metallic structures required for microelectronic devices. Researchers led by Michael Cullinan at the University of Texas at Austin have now developed a powerful process for 3D printing metal nanoparticles to generate precision structures with resolution below 5 microns. The authors demonstrate their microscale selective laser sintering (μ-SLS) technique in the context of producing lightweight lattice structures and metallic pillars that can serve as next-generation interconnects for microelectronics. Future iterations of μ-SLS could tackle even more aspects of the device fabrication process, or facilitate the production of specialized materials for plasmonics, microfluidics or other applications.

## Introduction

**Fig. 1:**
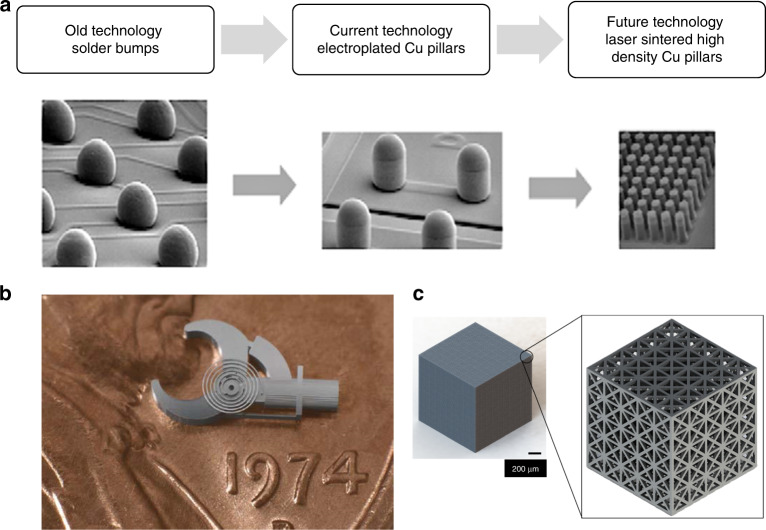
Potential applications of the μ-SLS process. **a** Evolution of C4 solder bumps(adapted from ref. ^33^)- The previous technology used ~1:1 aspect ratio SnAg bumps with >100 μm diameter features and >150 μm pitch. Current state-of-the-art technologies use sub-100 μm electroplated copper pillars to reduce the overall footprint of ICs. However, the process limitations of electroplating make it difficult to fabricate large-area, high aspect ratio sub-50 μm resolution structures. The μ-SLS process can enable the fabrication of the next generation of copper pillar-like structures with applications in flip-chip electronics industry. **b** Ni-Co compliant microforceps fabricated using a hybrid-AM technology useful in minimally invasive microsurgeries (adapted from ref. ^34^. Reproduced with permission from Emerald Publishing Limited). The high throughput capabilities of μ-SLS process can drive the production scale fabrication of essential microproducts like these. **c** A representation of large-area fabrication potential (1.2 × 1.2 × 1.2 mm^3^) of the μ-SLS process. Complex lattice-like microstructures (inset) have applications in developing extremely lightweight structural elements.

As the additive manufacturing (AM) technologies are maturing, more and more industries are looking into the possibilities of employing AM as part of their production ecosystems. In addition to the aerospace industry, which has been one of the earliest adopters and investors of the technology, newer avenues are being explored in the medical, transportation, consumer products, energy, tooling, art, jewelry, construction, and microelectronics industries^1–8^. With the current market value of over $7 billion, the growth of AM has been phenomenal over the last 8 years with an average annual growth of 27%^9,10^. While most of this growth has concentrated on macroscale AM tools and products, the continued demand for miniaturization of devices in the electronics, biotechnology, medical, automotive, and optics sectors is fueling increased interest in the development of microscale AM technologies as well^11,12^. However, there are significant challenges when scaling conventional AM down to the microscale including limited build rates, part complexities, and types of materials that can be used. In order to overcome these limitations, this paper presents a new microscale AM technique called microscale selective laser sintering (μ-SLS) that can produce true three-dimensional metal structures with a feature -size resolution of better than 5 μm. While the process has primary applications in microelectronics packaging, it can be used to fabricate microscale actuators, sensors, and micro-optoelectronic components as well. Therefore, future applications of this μ-SLS process will likely include the manufacturing of high performance designs of complex MEMS and medical devices (see Fig. 1b) which are difficult to fabricate using more conventional processes like Si-micromachining, electroplating, and etching^13–17^.

The current focus application of the μ-SLS process is the microelectronics packaging industry where the fabrication of microscale, 3D interconnect structures is becoming increasingly important. This is because the trend for increased miniaturization of electronic devices and the demand for higher performance devices with greater functionalities has resulted in the continual reduction in transistor size and more and more transistors getting packed into the same area on a chip^18,19^. However, there is a large disconnect between the current scaling trends in semiconductor chip manufacturing and the capabilities of the back-end-of-line fabrication processes used for packaging of these chips. For example, the solder bumps which have traditionally served as the interconnect between two dies or between the die and a substrate are traditionally manufactured through an electroplating process. As the solder bump process involves re-melting the solder balls, there is poor control over the morphology of the reflowed spheres. Fabricating these bumps below 125 μm pitch is difficult due to the decrease in standoff height and poorer joint reliability at smaller pitches. These often lead to device failure due to short-circuiting. To overcome these problems, more advanced electronics packages use copper pillar bumps which allow for finer pitches between interconnect structures and more precise gap control between the two surfaces being connected while maintaining sufficient standoff for mechanical strength. Additionally, Cu pillar bumps are also shown to have higher electrical and thermal conductivities and better electromigration reliability. As a result, pillar bumps are increasingly being adopted to replace solder bumps^20,21^. As we move to the next generation of these pillar bumps (from 90 μm pillar diameter/40 μm pillar height to 10 μm pillar diameter/50 μm pillar height—see Fig. 1a), the aspect ratio of the bumps keeps getting higher which is a problem since electroplating at this scale is limited to a maximum aspect ratio of ≈2:1^22^. Thus, fabricating these structures is out of the capabilities of the current electroplating processes used to make the pillar bump arrays.

One possible approach to make these structures is to additively manufacture these 3D interconnect structures. Unfortunately, none of the currently available microscale AM processes can produce these pillar-like structures with the rates, resolutions, and feature complexities required for the next generation of electronics packaging. For example, two photon lithography and interference lithography work only with polymers,^23,24^ so producing metal bumps would require some post processing steps, electrohydrodynamic jet printing and direct ink writing cannot produce the types of true 3D structures with overhangs that will be required for the next generation of 3D electronics^25–27^, and other processes such as electrochemical deposition and Laser Chemical Vapor deposition are too slow to be used in high volume production environments^28,29^. Overall, none of the existing microscale AM processes is capable of generating the types of true 3D metal structures with feature resolutions of less than 5 μm and volumetric throughputs greater than 50 mm^3^/hour that are required for the production-scale, microelectronics packaging environment. One process that holds promise for fabricating the desired metallic interconnect structures is selective laser sintering (SLS) since laser sintering is compatible with both metals and polymers and can generate 3D structures. However, the resolution of conventional macroscale SLS systems is limited to 70–100 μm due to the use of microscale powders (typically 5–20 μm) and laser spot sizes used in such systems^11,12,30^. μ-SLS overcomes these limitations in the macroscale SLS process and with other microscale AM processes by employing nanoscale particles as the parent material for sintering with additional focusing optics and precision motion systems that are used to achieve the desired resolution and high throughput.

Other potential applications of the μ-SLS process are in fabrication of ultra-lightweight structural members reinforced by lattice structures (See Fig. 1c) and novel interconnects for enabling three-dimensional (3D) integration in IC design. 3D integration is one of the most promising solutions to the problem of increasing interconnect delays due to increasing interconnect lengths created as the I/O density on chip increases. 3D integration can lead to a reduction in overall interconnect lengths, thus reducing time delays without increasing the footprint^31^. 3D packaging also allows for fabricating layers of dissimilar materials (metals and dielectrics), integrating different process technologies (memory, analog, rf) and functions on the chip^32^. Therefore, the ability of the μ-SLS process to fabricate complex, 3D interconnect structures could be a key enabling technology for the future of 3D electronics manufacturing. The rest of this paper presents an overview of the μ-SLS system and some preliminary results on fabricating microscale, 3D parts using this system.

## Overview of μ-SLS

**Fig. 2:**
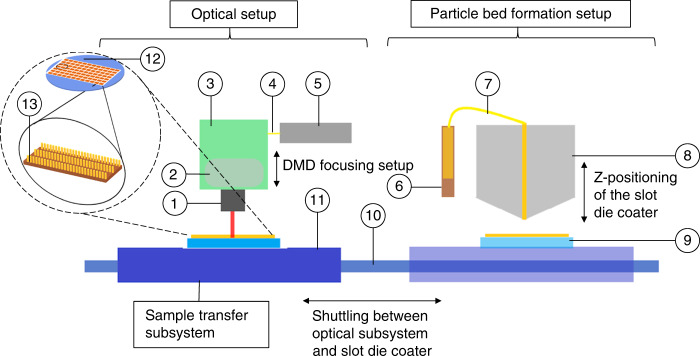
A simplified schematic of the μ-SLS process showing the different subsystems involved in layer-by-layer fabrication of a true-3D structure. Optical Setup. 1 – Secondary focusing optics. 2 – Primary beam-shaping optics for the Digital Micromirror Device (DMD) array. 3 – DMD array housing and controller. 4 – Optical fiber coupling the laser driver output and DMD array. 5 – CW Laser driver; Particle bed formation setup. 6 – Syringe pump for nanoparticle ink transfer into the slot die coater. 7 – Slot die coater tubing for fluid intake, aspiration, and distribution. 8 – Die head coupled with a vertical positioner for adjusting the coating gap in between layers. 9 – Heated vacuum chuck with the substrate/wafer; Sample Transfer Subsystem. 10 – Direct drive linear motor for shuttling the wafer between optical setup and slot die coating setup. 11 – XY-Nanopositioner for wafer alignment and stepping during large-area fabrication. 12 – A representation of wafer scale fabrication of the 3D parts. 13 – Enlarged view of the individual patterns formed by the μ-SLS process.

Figure 2 shows a schematic of the μ-SLS system. The system consists of three major sub-systems: (1) a particle bed formation (spreader) setup which is used to generate the sub-μm particle bed, (2) an optical setup which is comprised of laser system and optics designed to achieve the sintering resolution and high throughput^28^, and (3) a sample transfer, alignment, and metrology setup used to shuttle the particle bed between optical and spreader sub-systems with resolution of better than 10 nm. In addition, a vacuum chuck is used to hold the substrate in place and ensure that it does not deform during the coating or writing processes. The system sits on a vibration isolation stage to reduce outside influences that could damage the part quality.

The μ-SLS system also replaces the microscale powders used in conventional SLS processes with nanoparticle (NP) inks in order to achieve the desired feature resolution. To build layers that are ~1 μm thick, it is necessary to use particles that are at least one order of magnitude smaller than the desired layer thickness, hence the use of NPs in the system. The use of the NP ink also helps to prevent agglomeration of the NPs during the powder spreading process. The spreader mechanism employs a slot die coating system to spread uniform layers of NP inks over the substrate. The wafer holding vacuum chuck is equipped with heaters to reduce the thermal gradient across the sample and lower the energy requirements for the process in addition to securing the sample in place.

A one degree-of-freedom nanopositioning system with a resolution better than 200 nm is integrated in the slot die coating system to precisely control the coating gap and consequently, the thickness of the NP layer that is spread during the build process. In this system, an electromagnetic linear actuator is used to move the vacuum chuck under the slot die coater head and air bearings are used to guide the motion and ensure that a smooth, uniform coating is produced. The linear actuator is then used to move the sample (held on the chuck) to the optical station and is positioned under the optical system for sintering. In addition to the long travel linear motor/air bearing system, a flexure based nanopositioning system is used to precisely step the NP bed repeatedly under the optical station to pattern a much larger area in one pass under the optical station with a resolution of better than 10 nm.

The μ-SLS optical system consists of a continuous wave (CW)/quasi-CW laser coupled to an optical fiber and then directed off a micromirror array through a de-magnification optics setup. This allows each pixel in the DMD array to be focused down to a spot size of ≈1 μm^33^. Between sintering each layer, the height of the projection optics is adjusted to compensate for the change in powder bed height created by the spreading of the new NP layer. Once the wafer is patterned entirely, the sample is moved back to the coating station for next layer of NP coating and the process is repeated until the 3D part is complete. The final step in the process is removal of unsintered inks from the part which is achieved by ultrasonicating the sample in a solvent similar to the primary solvent of the ink for 30–60 s. The properties of the 3D part can be enhanced further by post processing of the sample. For example, annealing of the part after printing can be used to improve the part density and, hence, enhance electrical conductivity of the part, if required.

**Fig. 3:**
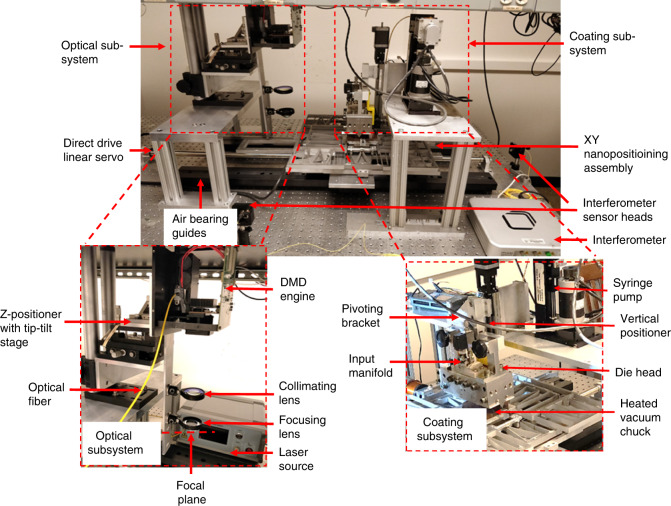
Physical embodiment of the μ-SLS system showing the optical and coating sub-systems along with the linear servomotor and air bearings used to transport the build substrate between the two sub-systems. An XY nanopositioning system with interferometric feedback sensors is used to precisely and repeatably position the build substrate under the optical sub-system for high speed patterning of each layer in the build process.

Figure 3 shows the actual physical embodiment of the μ-SLS system with the optical, coating, and positioning subsystems indicated. This embodiment of the μ-SLS is designed for the specific microelectronics packaging application described in the introduction section for 50 mm wafers. The throughput of the process is currently about 63 mm^3^/hour including the coating and sintering processes. However, the modular design of the system makes it easily adaptable for larger wafers and other applications if needed, with minor modifications in the sub-systems. The following sections of this paper describe each of the key specifics of the μ-SLS process in more detail and present some preliminary results for 3D microscale parts fabricated using this system.

## Process description

The three key steps in the μ-SLS process are: (1) NP bed formation to achieve sub-μm layer thicknesses with good packing density, consistency and repeatability, (2) Sintering of particles to achieve the desired sub-5 μm resolution and high throughput using the optical setup, and (3) High precision sample transfer, alignment motion systems, and metrology design for precise motion of the stage under the optical sub-system. How each of these process steps has been designed to achieve the desired resolution, throughput, and large area patterning will be discussed in the following sections.

### Nanoparticle bed formation

Achieving sub-5 μm feature resolution requires the use of particles that are at least an order of magnitude smaller than the desired feature resolution which results in the use of NPs in this system. The higher surface energies of NPs due to large surface area to volume ratio causes the NPs to agglomerate and form clusters^34–36^. Initial spreading results using nanoscale Cu powders confirmed these phenomena and showed the presence of large agglomerates in the bed of these nanoscale powders^37^. Regardless of the average particle size advertised by the manufacturer, the bed always contained agglomerates in the micron size range (see supplementary Fig. S1 for images of different NP samples). NPs also have higher a propensity towards oxidation^38–41^ which increases the energy required to sinter the NPs and in turn leads to insufficient sintering and thus, poor part quality. Effects of size and shape of the particles in the bed have also been shown to alter the particle packing efficiency in the bed and, thus, affect the layer densities and final part quality^42–44^. Based on preliminary testing of powder NPs, the powder samples were found to have a very wide size distribution, poor packing, irregular shapes and a high tendency to oxidize which prevent powder-based NPs from being used in the μ-SLS process. Therefore, a new power layer-by-layer NP coating process was devised using NP inks to create the multi-layer structures required by the μ-SLS process.

NP inks are NPs suspended in a dispersion typically with some surfactant coating (such as PVP or PEG) to prevent agglomeration of the NPs into clusters. The presence of an organic dispersant reduces the van-der-Waals interaction among the particles and presence of a protective surfactant coating reduces the extent of agglomeration in the sample^45^. The particles are found to be uniform in size and shape as opposed to the powders (see supplementary Fig. S1). Therefore, NP inks are ideal for use in the μ-SLS system. A detailed review of the effects of morphology and synthesis methods of NPs for use in micro-AM applications is presented by the authors in ref. ^37^. For the current generation of μ-SLS, commercial Ag (JSA102A, NovaCentrix, Austin, US) and Cu (CI005-G,Intrinsiq Materials, UK) NP inks have been tested and are being used in the tool.

The coating process for μ-SLS needs to be able to dispense uniform sub-micron layers of low viscosity NP inks to achieve near net shape features. A low material wastage process is always desirable from an economic point of view. Another desirable feature in the system is its compatibility with different substrates including flexible substrates and roll-to-roll flexibility so that it can be employed in the coming versions of the μ-SLS system. Many coating and printing techniques are available that can print thin layers of these inks such as spin coating, doctor blading, screen printing, slot-die coating and inkjet printing. Out of these, the slot die coating process provides very high uniformity of coating thickness, ranging from hundred nanometers to tens of microns^46–48^, and has the capability to spread multiple layers of NPs without disturbing the layers beneath it. It is a pre-metered coating process where the thickness of the coated layer is empirically dependent on process parameters such as the process speed, the die geometry, the ink flow rate, and the rheological properties of the liquid. These properties make slot die coating the optimal process of forming the build layers in μ-SLS.

The slot die coating setup consists of a vertical positioning mechanism actuated by a stepper motor, a cylindrical flexural bearing for in-plane positioning of the die head, and other support elements of the design. The fluid intake, aspiration, and distribution within the syringe pump is controlled using a three-way rotary valve. The slot die coater and the fluid lines are primed and purged of air bubbles before establishing the initial coating bead with the substrate. After the initial coating bead is spread uniformly across the width of the substrate, the NP inks are coated onto the substrate by translating the substrate under the slot die coater. The coated layer is partially dried in situ using the heated chuck to remove the excess solvent and facilitate the sintering process. To evaluate the fidelity of the coating process, the surface uniformity of a completely dried film was measured using an optical profilometer (Wyko NT 1100). Using this method, the average dried film thickness of the coated film when the substrate moved at a 10 mm/s speed with a constant coating gap of 100 μm was measured to be 520 ± 50 nm^49^. This corresponds to a ~1.5 micron thick wet layer thickness for a manufacturer specified 30% loading concentration of the NP ink. After sintering the first layer, the coating gap between the substrate and the die lips is increased using a vertical positioner and the process is repeated to lay down the next layer of NP ink without distorting the bottom layer. This coating system, therefore, allows sub-micron thick layers of NPs to be repeatably placed onto the substrate without disturbing the NP layers below the new coated layer.

### Laser sintering <5 μm features with high throughput

The μ-SLS system employs an optical system for printing the desired patterns onto the substrate with a feature resolution better than 5 μm and a pixel resolution close to 1 μm. A conventional macroscale SLS system uses a set of reflection mirrors, beam expanders, a 3-axis dynamic scanner or an *f*_*θ*_ lens and a galvanometric mirror for patterning the sample. However, as the desired feature resolution is roughly 100 times smaller than conventional SLS, the throughput of the process using the conventional optics will be reduced by a factor of 10,000. This makes the process unfeasible in an industrial setting for large scale microelectronics packaging. To increase the throughput of the process, a digital micromirror device (DMD) array from Texas Instruments (TI) is used in the μ-SLS system. A DMD is a micro-opto-electromechanical system used by TI and other companies for their projection systems. It is an array more than 2 million micro-scale mirrors that can be independently actuated/modulated between pre-set orientations. Using a DMD mirror for pattern generation, instead of sintering just one 1 μm spot at once, allows millions of 1 μm spots to be sintered at once thus greatly enhancing the areal throughput of the system.

A CW, a QCW or a nanosecond (NS) laser of appropriate power (typically 50 to 300 watts depending on the material being patterned and the layer thickness) is fiber-coupled to the micromirror array (DLP 6500) through a set of coupling optics that includes a fiber coupler, a beam expander, a fly’s eye lens, mirrors and lenses to resize, reshape and direct the input beam such that the laser beam fills the entire DMD array. The DLP 6500 chipset from TI contains a 1920 × 1080 array of micromirrors each of which is 7 μm in size separated by 600 nm distance apart. To get the desired 1 μm resolution of the system and achieve high throughput, the focusing optics is designed with the following requirements: (1) Achieve a magnification of 1/7 and (2) Collect the maximum possible light reflected out of the DMD setup in order to deliver the most possible power to the NPs. Maximum light collection is necessary for high fluence/irradiance at the sample plane which reduces the exposure duration, thus enhances the throughput while simultaneously reducing heat affected zones (HAZs). The optics design for the system is presented by the authors in ref. ^28^ and using this setup, an area of 2.3 mm by 1.3 mm can be patterned at once with a feature resolution of 1.2 μm which is close to the desired resolution. The optics are selected ensuring that the diffraction limited resolution of the focusing optics is smaller than the desired 1 μm pixel resolution.

**Fig. 4:**
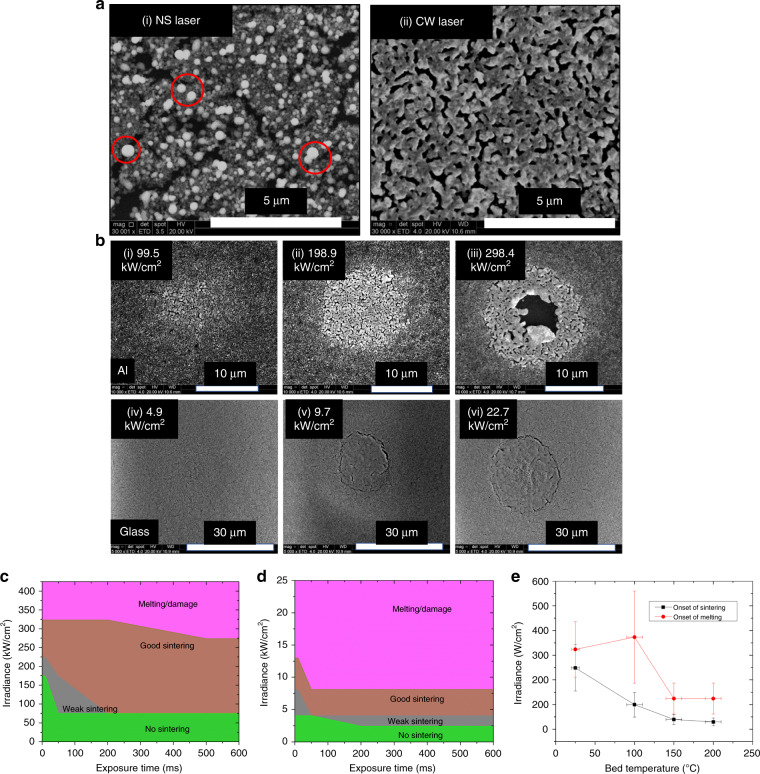
Comprehensive study of laser-material interaction for Cu nanoparticles. **a** Comparison of sintering quality using NS and CW lasers- (i) NS laser showing significant balling up effect (see red circled spots) with sporadic necking & crack developments (ii) CW laser showing no balling, uniform necking between particles throughout the spot area. **b** Variation in sintering quality of Cu NPs from no-sintering to weak sintering, good-sintering & melting as the irradiance of CW laser is increased for (i–iii) A 0.4 μm thick Cu layer on Al substrate for a 500 ms exposure duration (iv-vi) A 0.4 μm thick Cu layer on Glass substrate for 500 ms exposure. **c** A graph showing the laser irradiance vs exposure duration processing window for a 0.4um thick Cu NP layer on Al substrate. **d** Processing window for a 0.4 μm thick Cu NP layer on glass -substrate; experiments for Fig. 4a–d are conducted with particle bed at room temperature. **e** Reduction in sintering and melting onset threshold when using a heated bed at different initial temperatures.

A set of preliminary sintering experiments were conducted with different laser types including a femtosecond (FS), a NS, and a CW laser to compare the quality of sintered regions obtained with different laser types. Based on those experiments, it was found that both FS and NS laser sintered spots showed significant balling up of NPs (with balled up agglomerates of sizes ranging from 500 nm to 1 μm—see the areas circled in Fig. 4a) which leads to poor bed density in the final part and hence, poor properties of the sintered part^50,51^. In comparison, CW laser sintering showed a better sintering quality as the balling up of NPs effect was negligible and the necking between the particles was uniform throughout the spot area (see Fig. 4). The average power requirements using CW laser are typically much higher than average power requirements for short-pulsed (NS and FS) lasers due to the enhanced heat transfer away from the laser spot for the longer exposure, CW laser. This lower average power during sintering with the short-pulsed lasers results in a processing window that is very narrow in terms of laser power that can be used for getting good sintering as compared to the CW laser. As a result of this narrow processing window and local spatial variations in the NP bed/laser beam intensity, localized melt pools can form during the sintering process with the NS and FS lasers which results in formation of balls in the powder bed due to the hydrodynamic effects (minimize the surface energy) of the liquid metal on the substrate. The size of these balls typically ranges from a few 100 nm up to 1 μm depending upon the average size of NPs that are melted. Figure 4a, b shows a comparison of the sintering quality of spots sintered with laser powers tuned for good sintering quality using NS laser and CW laser. A 532 nm green NS laser with a pulse duration of 25 ns and a 532 nm CW laser were used for the sintering of these spots. Figure 4c–e provides a summary of the exhaustive laser sintering study carried out by the authors to obtain the processing window for good sintering at different laser powers, exposure durations and bed temperatures. For further details regarding the experiment design, procedure and analysis of these results, readers are encouraged to review the following article by the authors^51^.

A shortcoming of laser-based processes is the development of large thermal gradients leading to residual stresses, deformations and distortions in the part. Powder beds in conventional laser sintering are typically heated to a high temperature before exposure to the laser which helps to minimize the thermal gradients between the un-sintered and sintered sections of the bed. This helps to reduce the development of high residual stresses and hence, improves part quality. In addition to the improvement in final part’s properties and lower distortions, heating the particle bed also lowers the energy requirements for the sintering process. This has been confirmed by sintering experiments conducted by the authors using a heated stage for wafer handling in ref. ^50^. The laser energy requirements were found to decrease by up to 17 times when the bed was heated to 200 °C compared to the energy requirements when the bed is at room temperature^50^ (see Fig. 4e). Thus, a wafer handling chuck with heating capabilities is used which reduces the wattage requirement of the laser and leads to better quality parts with smaller geometrical aberrations and less warpage (see supplementary Fig. S2 for the design of this chuck)^52^.

### Sample transfer and alignment

There are two sub-systems included in the sample transfer and alignment process: (1) a long-range linear motion system used to shuttle the sample between the slot die coating and optical system and (2) an XY nanopositioning stage used to align the sample under the optical system for minimizing registration errors and move the sample to sinter the whole 50 mm by 50 mm sample in one pass under the optical sub-system.

#### Long-range linear motion between spreader and sintering stations

To achieve 1 μm feature resolution, the registration errors between two consecutive layers of NPs should be minimum, preferably <100 nm so that even after 10 layers, the errors are within 1 μm pixel resolution specification. The motion between subsystems should also be highly repeatable which requires that the non-linear effects commonly associated with contact-based bearings such as stick slip phenomenon and friction effects should be minimized. Air bearings are advantageous compared to rolling element bearings due to frictionless operation (minimal air shear friction at high speeds), minimum wear, high speed capability, and no requirement of lubrication in the system which also means lower impact on the environment. Since the friction in air bearings is dependent on air shear from motion, the friction when starting (at zero velocity) is zero and hence, there is no stiction in the system allowing it to have a high resolution and repeatability. In this μ-SLS system, we are using an air bearing system from NewWay Air bearings. The system consists of two, 1 m long, parallel rails as guides for the two trucks which hold the nanopositioning stage. The air bearing stage is actuated using a direct drive linear servo motor which is also non-contact and requires no mechanical linkages/mechanisms for motion transmission, thus minimizing potential sources of friction and non-linearities in the system. For position sensing of the center stage along the direction of motion, an interferometer (model: FPS3010, from Attocube Systems AG) with a resolution of 1 pm and a working range of 2 m is used.

#### High precision alignment using XY nanopositioner

A nanopositioning stage is designed to align the sample accurately and precisely under the optical system and move the sample in a step-and-repeat fashion to sinter multiple patterns in one go under the optical system. Once the sample reaches the optical sub-system, the next step is to align the sample under the optical sub-system accurately with good repeatability so that the overlay errors are minimized. A motion resolution <100 nm ensures that the overlay errors even after 10 layers stays within the desired feature resolution on the part. Secondly, the optical sub-system design patterns an area of 2.3 mm by 1.3 mm in one shot whereas, the size of each wafer is 50 mm by 50 mm. Thus, the nanopositioner needs to have a travel range of 50 mm in both the x and y directions so that the entire wafer can be patterned in one pass under the optical sub-system. The wafers are patterned using step and repeat approach where the whole wafer will be composed of multiple exposures (each exposure sized 2.3 mm × 1.3 mm). Thirdly, to maximize the throughput of the system, the idle times while sintering should be minimized. Using a 50 ms exposure duration^43^ under the optical sub-system for sintering and another 50 ms for moving between steps and achieving steady state, the motion system is desired to operate at speeds of 10 Hz which requires that any uncontrollable resonances of the motion system should be at greater than 30 Hz so as to not affect the motion quality of the stage at 10 Hz stepping. And lastly, the motion system is desired to have a repeatability of better than 50 nm. To achieve this repeatability, effects such as friction and backlash in the system need to be minimized. Although, a number of off the shelf nanopositioning stages are available that can achieve a resolution of less than 5 nm, most of them are piezo-actuated stages and hence, are limited in their range to a few hundred microns. The traditional XY stages based on rolling element bearings can achieve the 50 mm travel but their motion quality is poor due to friction and stiction in the system. Air bearings and magnetic bearings driven by direct drive linear servos can achieve both the resolution and range requirements, but they are not cost effective. Thus, a flexure bearing stage driven by voice coil actuators are used as flexure bearings provide a low-cost, high motion quality, and easy to design solution.

The stage design makes use of a modified double parallelogram flexure mechanism (DPFM) as its building block and these modified DPFMs^53^ are connected in parallel to achieve the desired stiffness and travel range for each axis. The stage uses a modular two-level stage design using 12 modified DPFM units with 6 units guiding the motion along each axis. The modified DPFM units are waterjet cut out from ½” thick Al 7075 plates and assembled. Details on the design of the modified DPFM stage and its assembly can be found in other works by the authors^54–56^. For actuation, two sets of voice coil actuators are used to drive the bearings and a set of interferometers are used for position sensing and feedback control of the center stage. The closed loop positioning resolution is of this stage is found to be 7.6 nm at a 13.7 Hz bandwidth. The details of the controller design and its tracking performance are given in ref. ^56^. Overall, the stage is shown to achieve three very important specifications: (1) it has been shown to have a sub 10-nm resolution, (2) it has a tracking bandwidth of greater than 10 Hz, and (3) it has a travel range of over 50 mm in both the *x* and *y* axis. Therefore, when this stage is combined with the long-range linear motion system described in the previous subsection, it is able to meet all of the motion requirements of the μ-SLS system.

## μ- SLS process flow

**Fig. 5:**
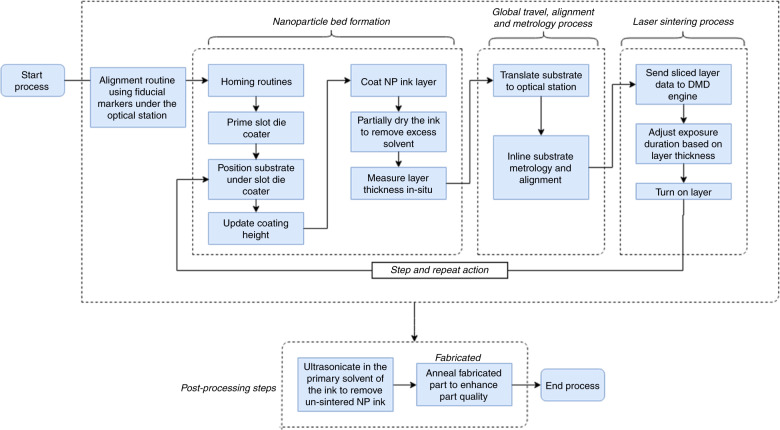
μ-SLS process flow-chart. The process starts with a homing routine for the initial optical alignment of the sample under the optical station using fiducial markers. The slot-die coater and the plumbing lines are primed and the sample is then translated to the slot die coating system where the nanoparticle ink bed is laid down. The ink is partially dried using an infrared heater and the layer height is measured in situ. The sample is then moved under the optical system where the initial position alignment is done. After the position is fixed, the sliced layer pattern of the 3D CAD model is sent to the DMD controller and the pattern is projected on the coated layer during the laser sintering process. The XY-Nanopositioner is stepped and the pattern is scanned over the sample area. The sample is then translated back to the slot die coating station where the next layer is laid down, and after which the sintering process is repeated. To maintain layer overlay control, the XY-nanopositioner is used to align the layers. After n layers are sintered, the excess NP ink is removed using a sonicator and the fabricated part is annealed to ensure good part quality.

Figure 5 summarizes the process steps for the μ-SLS process. The steps are listed as follows:

1. The first step in the process is the alignment routine to determine and set the XY coordinates for optical station and coating station. The samples have fiducial markers on the substrate and the alignment is done using an optical metrology system and the corresponding XY positions for coating and sintering locations (using interferometer) are stored in the memory. Any long-range motion of the sample is achieved through the linear motor-air bearing combination and XY nanopositioner is used for short range fine positioning.
2. Next, for spreading the NP layer, the slot die coater goes through its homing and priming routines to establish a uniform bead thickness for the coating. The sample is brought to the pre-determined XY location for coating (from step 1) and the z-gap between the sample and die lips is measured using the Keyence gap sensor. The sensor serves as a feedback for the z-motion control of slot die head to achieve the desired coating thickness.
3. Once the first NP layer is spread, the sample is heated using thermoelectric heaters and a heat lamp source to partially dry the wet layer. As the layer is partially dried, it is shuttled to the optical station on the air bearing guides using the feedback from the interferometers. The sample is positioned to the XY location for sintering (from step 1). The XY fine alignment is completed using the XY nanopositioner.
4. The 3D CAD of the final part is sliced into cross-sectional layers and this layer profile is fed into the DMD setup for turning the mirrors ‘on’ in the DMD array selectively. The thermoelectric heating is kept on to achieve a higher bed temperature and the laser exposure duration for sintering is modulated based on the layer thickness to achieve good sintering quality. Each exposure sinters a 2.3 mm × 1.3 mm area respectively. To sinter the whole 50 mm by 50 mm wafer, the sample is stepped and aligned using the XY nanopositioner and roughly 800 exposures are needed to sinter the entire wafer.
5. This whole procedure (steps 2–4) is repeated for each additional layer in the 3D structure until the desired part geometry is complete. Once the part is complete, the sample is brought to the washing station for removal of the un-sintered NPs from the part. This washing off procedure involves ultrasonicating the part in the parent ink solvent such that the washed off ink can be recycled and reused in the process after some chemical processing.
6. Finally, the part is annealed in an oven for a fixed time duration to enhance its electrical and mechanical properties.

There is a continuous need to economically evaluate and predict the quality of the products made using this microscale AM process. Understanding the physics behind laser-material interaction and NP sintering is critical to the fabrication of defect-free parts. Multiscale computational models for NP bed formation and consolidation are being developed, which will help in optimizing the process parameters required for creating complex microparts^57,58^. Additionally, these models will be calibrated against experimental results to improve their reliability in predicting sintered part.

## Sintering results

**Fig. 6:**
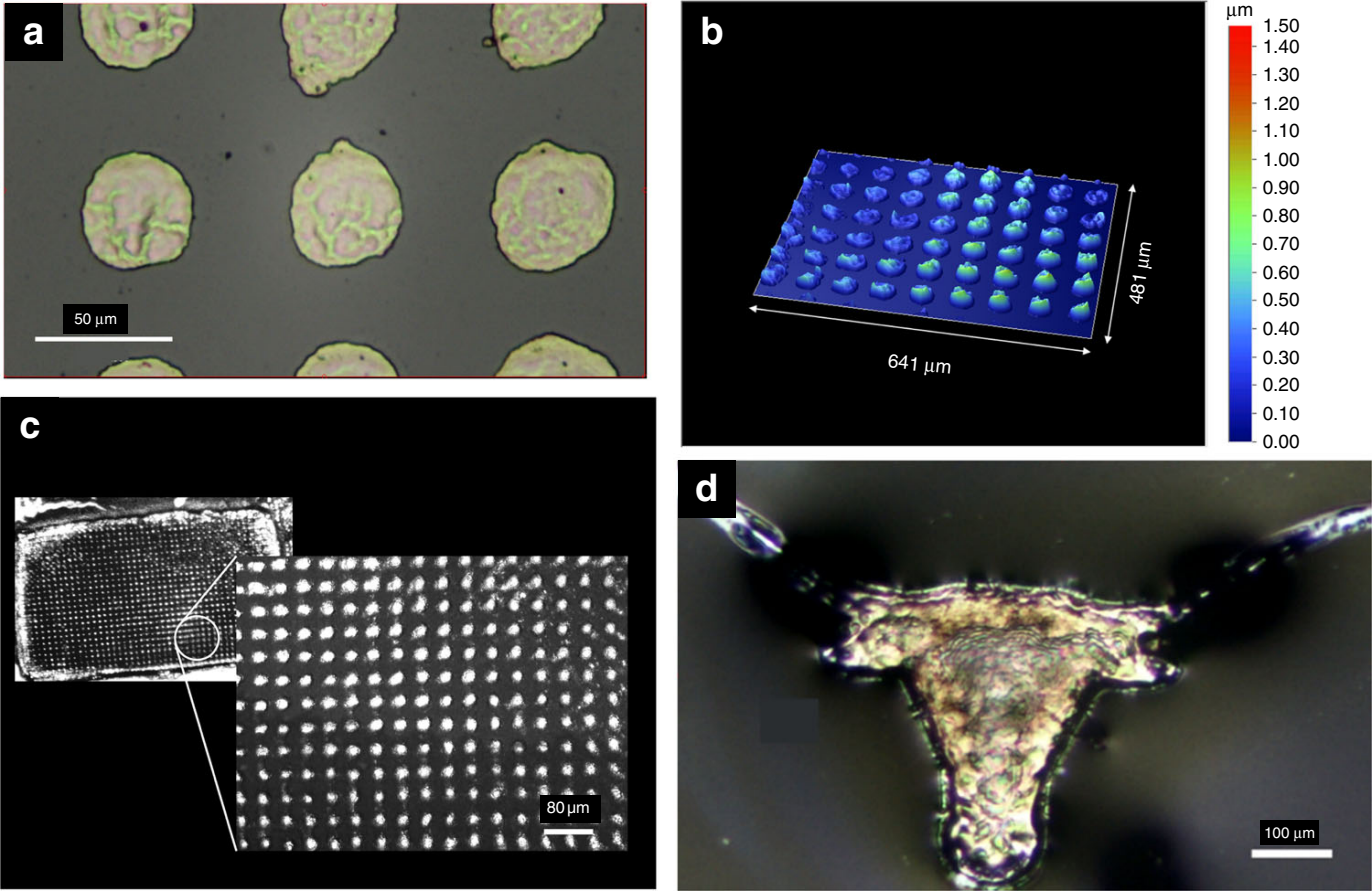
Single layer sintering results with Ag nanoparticle ink. **a** Optical microscope image of an array of 40 μm diameter circles with 80 μm pitch. **b** Surface topography of an array of 40 μm diameter circles with 80 μm pitch. The RMS height of these circles is around 617 nm with a standard deviation of 119 nm. **c** An optical microscope image showing the full areal scale (2.3 mm × 1.2 mm) array sintered on a 1 μm thick Ag NP ink layer. (inset) Enlarged image of an array of 20 μm diameter circles with 40 μm pitch. **d** Optical microscope image of longhorn logo (trademark of The University of Texas at Austin). The smallest feature size in this sintered part is around 7 μm near the tip of the longhorn ears.

With the design and assembly of the system complete, some preliminary testing was conducted to verify the resolution of the system. A 50 W CW laser with a central wavelength of 808 nm was used as the source. Shown below are some examples of the sintered patterns (un-sintered portion washed off) using the μ-SLS system. Figure 6a–c shows the optical microscope and surface profile images of different desired diameter circles that demonstrate the capability of the system to produce 3D pillar structures for IC packaging with diameters down to 10 μm (supplementary Fig. S3) using the current laser source over large areas. Figure 6d shows UT Austin trademarked longhorn logo, where the minimum feature size is around 7 μm. The sintering resolution of the system has been demonstrated to be close to 3 μm with the current setup (see supplementary Fig. S3). The overall resolution of these parts is affected as the higher exposure durations (>3 s) leads to the formation of HAZs around the feature boundaries. This effect may be attenuated by using a higher power laser source or even using a Quasi-CW laser source such that the exposure durations are much shorter thus reducing the heat propagation and minimizing HAZs. It is expected that reducing the HAZs should further improve the resolution of these structures bringing it closer to the optical resolution of the setup. However, these feature dimensions are already an improvement over the current electroplating technology used for fabrication of pillar bumps used in semiconductor packaging.

**Fig. 7:**
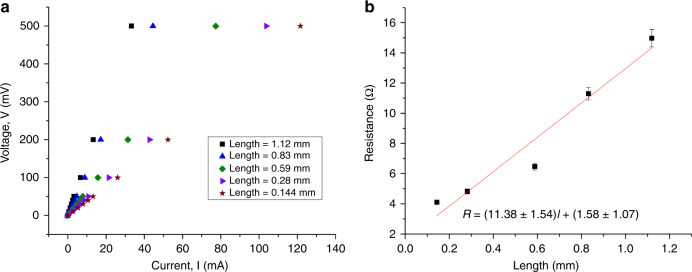
**a** V–I characteristic of a sintered and annealed @ 150 °C for 30 min Ag pattern for different lengths of the conductor. **b** Resistance, R vs Length, L curve for sintered and post-annealed Ag line.

Measuring the electrical resistivity of the sintered samples provides a convenient and accurate metric for assessing the quality of sintering using the μ-SLS system. The resistivity was measured using a four-point probe method. For these measurements, 80 μm wide lines with a thickness of about 80 nm are sintered and the V–I characteristics are obtained for different lengths of sintered lines (see Fig. 6). Assuming a linear relationship between the resistance (R) and length (L) of the conductor, the gradient of the R vs. L curve is the ratio of bulk resistivity and area of cross section of the conductor. V–I curves for different lengths of the conductor are shown in Fig. 6a and the resistance vs. length plot is shown in Fig. 7b. These results were collected from three sets of measurements with multiple samples at same lengths of the conductor. Using the slope of the fitting, the resistivity of the sintered samples is measured to be 73 ± 10 nΩ m. This is about 4.5 times the bulk resistivity of silver (16 nΩ m @ 20 °C) which is similar to or even slightly better than the conductivity of sintered silver reported in literature^59–61^. Good electrical conductivity of the sintered lines is critical for application of the process in semiconductor packaging applications.

### Multi-layer sintering results

**Fig. 8:**
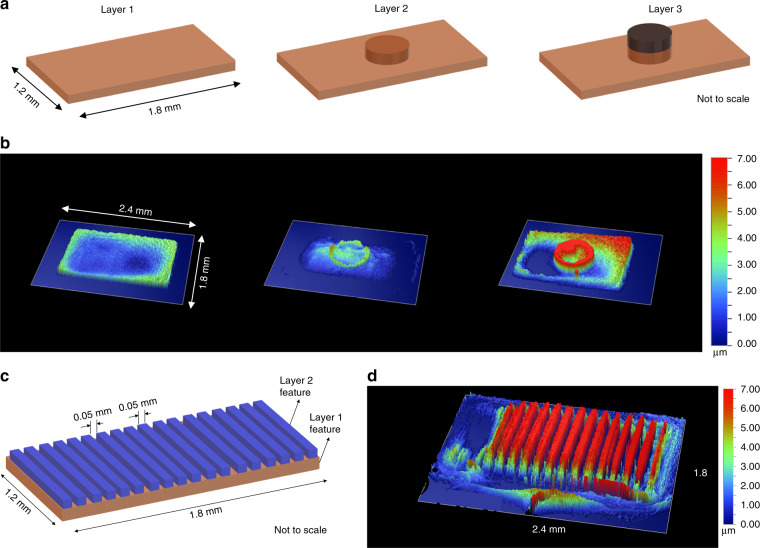
Multilayer sintering of Ag nanoparticle inks. **a** Visualization of the layer-by-layer (LbL) fabrication. The first layer is a 1.8 mm by 1.2 mm rectangular structure. A 500 μm circle is sintered on top of the first layer as part of the second and third layer. **b** Optical topographic images showing the LbL progression of the µ-SLS process. The RMS surface roughness are 2.24 μm, 1.19 μm and 4.08 μm Layers 1, 2 and 3 respectively. **c** Visualization of the two layer part with the first layer as a rectangular base and the second layer as a linear array. **d** Surface topography of the sintered and post-processed two-layer sample. The RMS height of the rectangular pillars measured from the top of layer 1 is 5.77 μm with a standard deviation of 1.75 μm. The averaged variability in pillar heights across the length of the array is 1.2 μm. These variations can be attributed to the non-uniformity in the layer deposition process, evolution of thermal gradients due to long exposure durations and the build-up of excess ink along the edges of the features during the sintering process.

To test the ability of the system for sintering multilayer structures with good overlay, several multilayer structures were fabricated. Figure 8a shows the progression of the feature heights as higher layers are coated. A 1.8 mm × 1.2 mm rectangular feature is sintered on the first coated layer to distinguish it from the subsequent layers. The average height of the feature is 2.24 μm. Afterwards, the next layers are coated and partially dried and a 500 μm circle is sintered. The average height of the top plane of the circle from the reference on the substrate is 3.98 μm for bilayer sintering and 8.02 μm for three-layer sintering. The coating gap between the die lips and the current layer is kept the same at 100 μm for the experiment. However, a sharp increase in the height of the part as shown in Fig. 8b can be attributed to the flow of excess ink into the sintering region, thereby affecting the overall uniformity of the part. Figure 8d shows a two-layer sintered part with the first layer being a rectangular base and the second layer is a linear array with 50 μm line width and 50 μm spacing.

Overall, these results demonstrate the ability of the μ-SLS system to preform, multilayer 3D part fabrications. However, better process tuning for different sub-systems such as optimizing the slot die coating parameters for better layer thickness uniformity and repeatability, improving the optics for better alignment and smaller spherical aberrations, developing DMD pattern correction schemes for better feature resolutions, and optimizing the sintering irradiance vs exposure duration for different layer thicknesses are some of the short-term future work that need to be completed in order to improve the quality, uniformity, and resolution of the parts produced.

## Conclusion

This paper presents a new micro-AM process called micro-scale selective laser sintering (μ-SLS) that can be used to fabricate three-dimensional parts with feature resolutions of better than 5 μm with a high throughput that can be employed in a production environment. As the different sub-systems of the technology mature, it is expected that the technology could eventually be employed in fabrication of complete microelectronic packages through chip-to-chip and chip-to-substrate interconnects, thus replacing the conventional building blocks of packaging including inter-layer vias, controlled collapse chip connection (C4) bump pads, ball grid array (BGA) pads, and flip chip metallic pillar bumps thus providing a complete solution for the back end-of-line assembly for electronics packaging. Additionally, the modular nature of the process allows for modifications in individual sub-systems for use in roll-to-roll applications for flexible electronics or in other micro-AM applications of metals such as the fabrication of metamaterials with desirable optical and mechanical properties, MEMS, plasmonics, hierarchical materials, and microfluidics^62–68^. Overall, this paper demonstrates that μ-SLS has the long-term potential help overcome many of the current limitations in microscale AM of 3D metal parts.

## Supplementary information

Supplementary Information for ‘A Novel Microscale Selective Laser Sintering (μ-SLS) Process for the Fabrication of Mircoelectronic Parts’

## Data Availability

The data (profilometer and SEM images) generated in this study are included in the manuscript. Any other data or details can be made available from the corresponding author on reasonable request.
