# Supplementary material for: A novel microscale selective laser sintering (μ-SLS) process for the fabrication of microelectronic parts
Source: Microsyst Nanoeng. 2019 Dec 30;5:64. doi: 10.1038/s41378-019-0116-8 (PMC8433322; doi:10.1038/s41378-019-0116-8)
Supplement: Supplementary file 1 — Supplementary Information for ‘A Novel Microscale Selective Laser Sintering (μ-SLS) Process for the Fabrication of Mircoelectronic Parts’ [file 41378_2019_116_MOESM1_ESM.docx]

**Supplementary Information for ‘A Novel Microscale Selective Laser Sintering (μ-SLS) Process for the Fabrication of 3D Electronic Parts’**


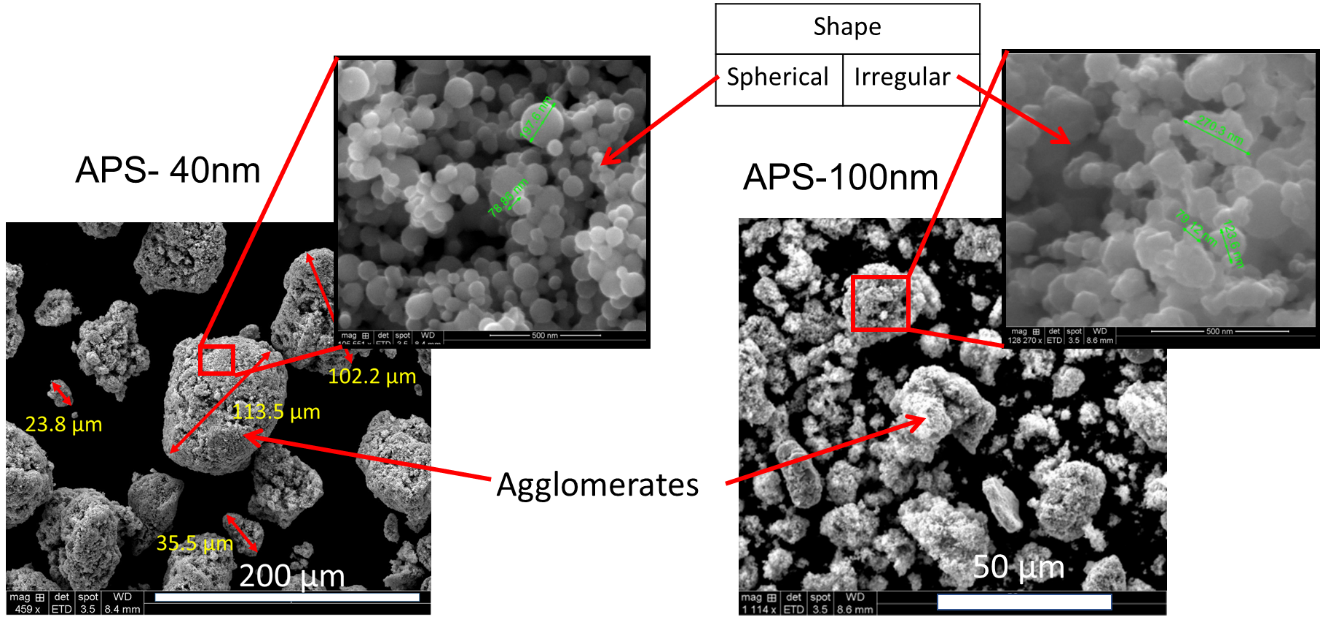


| (a) | (b) |
| --- | --- |


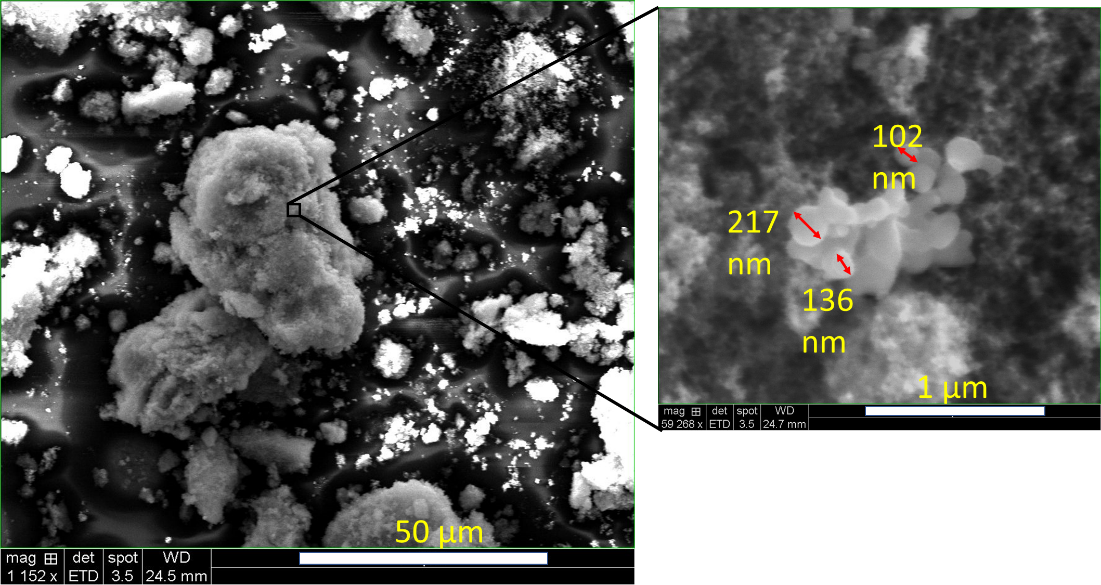


(c)


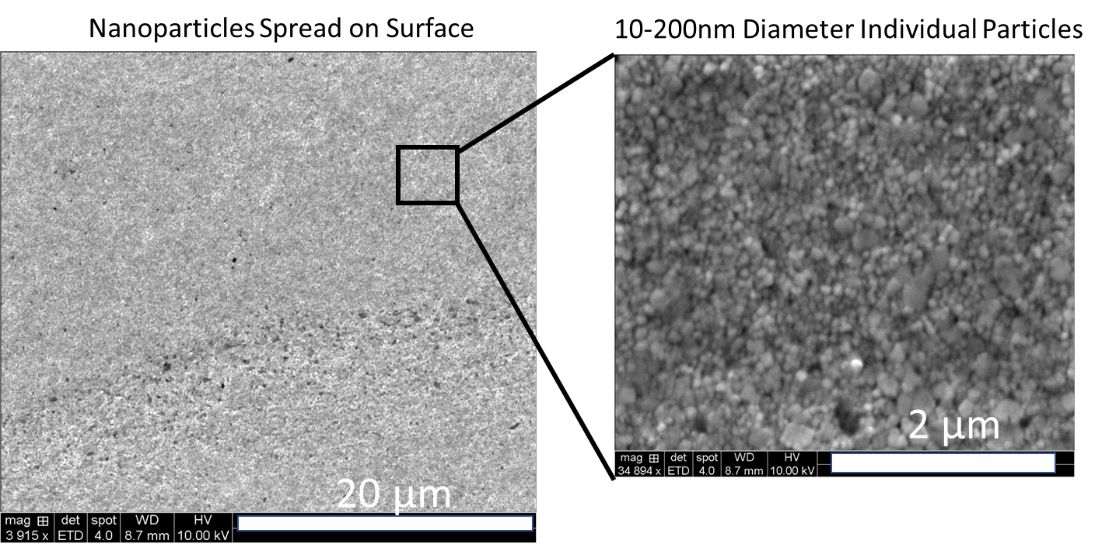


(d)

*Figure S1. a) SEM image showing nanoparticle agglomerates as big as 115 μm in a Cu Nanopowder sample with average particle size (APS) of 40 nm specified by the vendor (US Research Nanomaterials, Inc) & no coating , the inset SEM image showing the variation in size of the nanoparticles in the powder sample b) SEM image showing agglomeration of nanoparticles in another Cu NP with APS- 100 nm (no coating) as specified by the vendor(US Research Nanomaterials, Inc) - inset image showing variation in particle size and irregular shape of the NPs c) SEM images of carbon-coated 25 nm APS Cu NPs (US Research Nanomaterials, Inc) d) SEM image showing the uniformity & lack of agglomerates in a spin coated layer of Cu NP ink, inset image showing the uniformity & packing of nanoparticles in the bed (Applied Nanotech, Inc)*


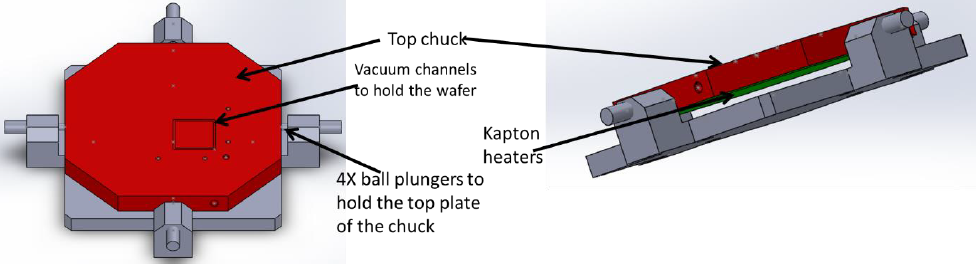


*Figure S2. Cartoon of the sample holding chuck for μ-SLS system- the chuck is designed using a Te-Cu Alloy with a 25% lower CTE than Cu and the design is symmetric & constrained at the four point contacts at the sides which allow for the plate to expand while heating & minimize the warpage of the chuck in the z-direction*


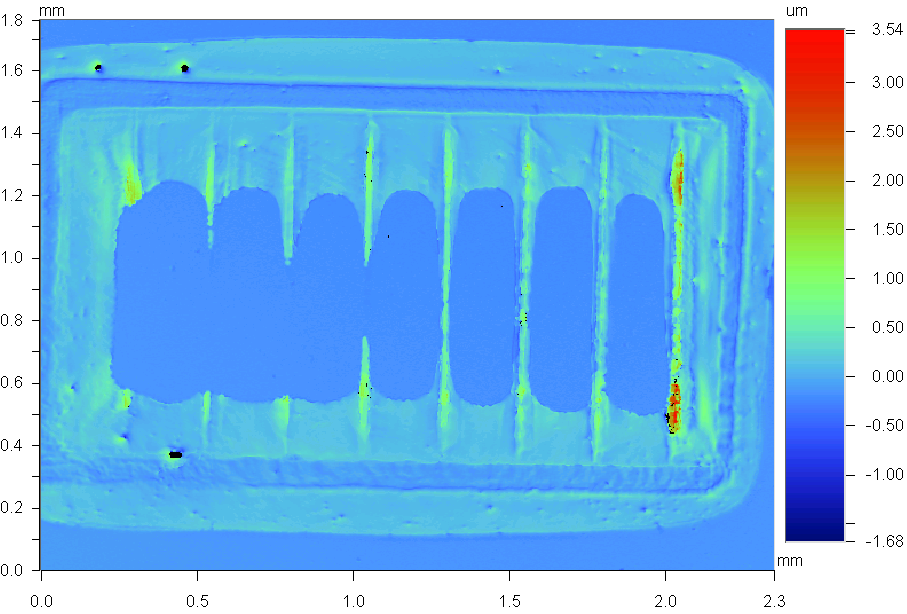

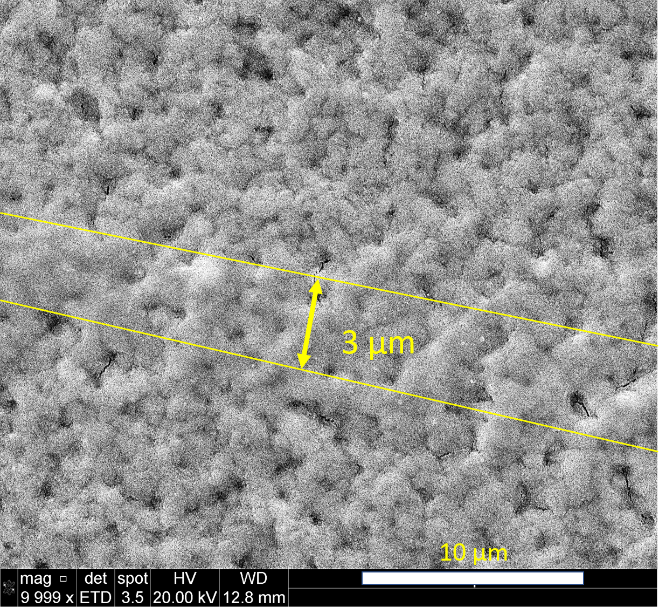


| (a) | (b) |
| --- | --- |


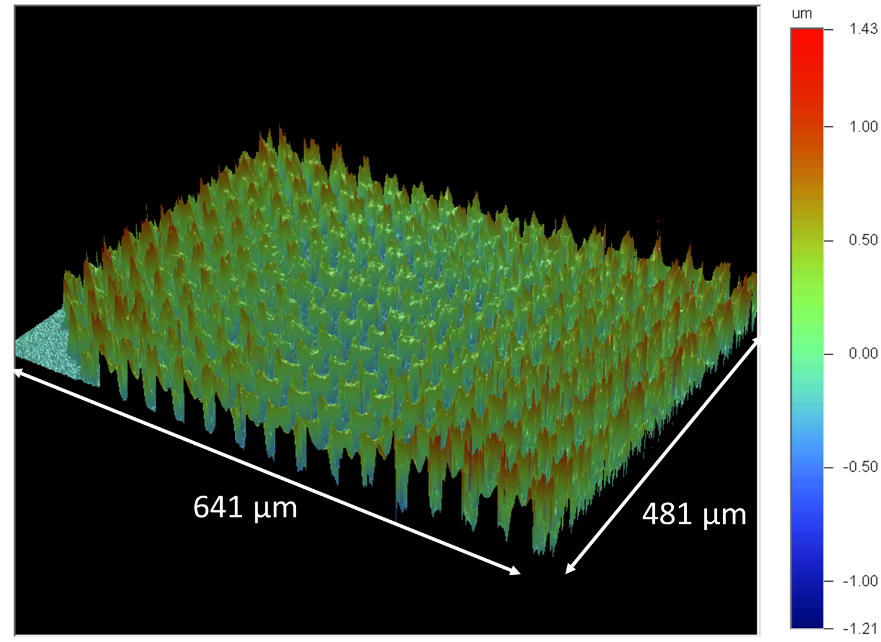


(c)

*Figure S3. Surface topography of a sintered rectangular pattern with periodic lines of uniform thickness and spacing, surrounded by 80 μm wide boundary (sintered and washed off unsintered portions)- 5 μm line width, 200 μm line spacing with an exposure duration of 10 s (b) sintered (unwashed) SEM image of a sintered pattern (10 s exposure) with 3 μm wide lines (c) Surface topography of an array of 10 μm diameter circles and a 40 μm pitch*
